# The role of a student-run clinic in providing primary care for Calgary’s homeless populations: a qualitative study

**DOI:** 10.1186/1472-6963-13-277

**Published:** 2013-07-17

**Authors:** David JT Campbell, Katherine Gibson, Braden G O’Neill, Wilfreda E Thurston

**Affiliations:** 1 Department of Community Health Sciences, Faculty of Medicine, University of Calgary, Calgary, Alberta, Canada; 2 Department of Medicine, Faculty of Medicine, University of Calgary, Calgary, Alberta, Canada; 3 Department of Family Medicine, Faculty of Medicine, Queens University, Kingston, Ontario, Canada; 4 Department of Primary Care Health Sciences, Medical Sciences Division, University of Oxford, Oxford, Oxfordshire, UK; 5 Undergraduate Medical Education, Faculty of Medicine, University of Calgary, Calgary, Alberta, Canada; 6 Department of Ecosystem and Public Health, Faculty of Veterinary Medicine, University of Calgary, Calgary, Alberta, Canada

**Keywords:** Primary care, Homeless persons, Medical student, Physician shortage areas

## Abstract

**Background:**

Despite the increasing popularity of Student-Run Clinics (SRCs) in Canada, there is little existing literature exploring their role within the Canadian healthcare system. Generalizing American literature to Canadian SRCs is inappropriate, given significant differences in healthcare delivery between the two countries. Medical students at the University of Calgary started a SRC serving Calgary’s homeless population at the Calgary Drop-In and Rehabilitation Centre (CDIRC). This study explored stakeholders’ desired role for a SRC within Calgary’s primary healthcare system and potential barriers it may face.

**Methods:**

Individual and group semi-structured interviews were undertaken with key stakeholders in the SRC project: clients (potential patients), CDIRC staff, staff from other stakeholder organizations, medical students, and faculty members. Convenience sampling was used in the recruitment of client participants. Interview transcripts were analyzed using a coding template which was derived from the literature.

**Results:**

Participants identified factors related to the clinic and to medical students that suggest there is an important role for a SRC in Calgary. The clinic was cited as improving access to primary healthcare for individuals experiencing homelessness. It was suggested that students may be ideally suited to provide empathetic healthcare to this population. Barriers to success were identified, including continuity of care and the exclusion of some subsets of the homeless population due to location.

**Conclusions:**

SRCs possess several unique features that may make them a potentially important primary healthcare resource for the homeless. Participants identified numerous benefits of the SRC to providing primary care for homeless individuals, as well as several important limitations that need to be accounted for when designing and implementing such a program.

## Background

Student Run Clinics (SRCs) are increasingly important contributors to healthcare in North America. There are over 100 SRCs associated with medical schools in the United States [1]. In Canada, there are eight SRCs, affiliated with the Universities of British Columbia, Alberta, Calgary, Saskatchewan (x2), Manitoba, and Toronto [2]. SRCs most often provide primary care services to the socioeconomically disadvantaged and underserved [3] who often experience worse health outcomes than the general population [4]. Medical students provide the majority of care in these settings, although there are significant contributions from students in other health professions [5].

Although there is extensive literature on how SRCs in the United States meet health needs, there is a paucity of literature on their Canadian counterparts. The substantial differences between healthcare systems in Canada and the United States [6,7] mean that SRC services meet a need of healthcare accessibility in the United States that is markedly different from that in Canada. Since program planning is dependent on systematic research within the local context [8] it is necessary to study SRCs in Canada to understand their potential role.

There are many purported benefits of SRCs for patients, through the provision of high quality care for patients who would not otherwise have access to necessary healthcare [9]. The quality of care provided by SRCs has been shown to be very high across a variety of conditions, including in mental health care [10] and diabetes care [11]. In the United States, SRCs provide an opportunity to assist uninsured patients to obtain insurance, an important step in improving the quality and accessibility of healthcare for this population [12].

Patients are very satisfied with the care they receive at student-run clinics [1,9]; which meets an important ethical imperative that students’ education not take precedence over the provision of high quality clinical care to clinic patients [13]. Those who are seen at SRCs have poorer health status than the general population [14], highlighting the importance of caring for this segment of the population.

It has also been shown that SRCs can be appropriate and beneficial adjuncts to clinical medical education [5,15,16]. The benefits are wide-ranging and include opportunities to engage in planning health education programs and working in an interprofessional environment [3,17] as well as the development of heightened social awareness [18]. Students who participate in a SRC have been shown to be more likely to go on to selecting primary care specialties [19,20]; given the urgent need for primary care practitioners in the United States and Canada [21,22] and the concomitant decline in graduates choosing primary care as a specialty [23,24] this represents a significant potential benefit to healthcare systems and to patients.

### The Calgary student-Run clinic

In January 2010, medical students from the University of Calgary opened a SRC at the Calgary Drop-In and Rehabilitation Centre (CDIRC), Canada’s largest homeless shelter. During weekly evening clinic hours, a team of two students see patients. They take a history and perform an initial physical examination that is reviewed with the attending physician, and together they develop a plan for the patient’s care. General primary care is provided to whomever requests clinic services. And as is commonplace in the Canadian health care system, there is no discrimination on the basis of payment. In contrast to other clinics, however, patients are seen in the Calgary SRC free of charge, even if they do not have proof of government health insurance. In addition to providing these services, students also assist patients with logistical arrangements for specialist or supportive care, and provide assistance obtaining relevant insurance or emergency medication payment if required. Clinic scheduling is arranged so that there is always one person present in the clinic on any given week who was also present the prior week; this facilitates continuity of care.

Our study purpose was to describe what the SRC could contribute to care for homeless individuals in Calgary, and to describe what success would mean for various stakeholders.

## Methods

The study design was grounded in program planning and evaluation theory, particularly utilization-focused evaluation with its aim of program and policy development [25]. Assessment of the actual and perceived role of the clinic was considered important in developing program policies for the SRC and before further research, such as studies of outcomes, can be undertaken [25]. In addition, assessing the clarity of the goals and expectations of the SRC among stakeholders was considered essential [8,26,27]. Qualitative methods were chosen, as they allow for the richest exploration of stakeholder perceptions [28]. Data collection methods included individual and group interviews.

Key stakeholder groups, identified as those with an immediate influence on the future of the SRC, were decided by the research team. Numbers of subjects were decided a priori, based on coverage of the key organizations and what was permitted by our funding: homeless clients (potential patients) (n = 11); CDIRC staff (n = 5); medical students (potential future SRC volunteers) (n = 6); faculty members (who were personally involved in the development and application of University medical education policy) (n = 4); and leaders from other agencies providing services to the homeless (n = 8). Purposive sampling [29] was used in recruiting participants. CDIRC staff were selected based on their positions, with representation from the core medical team, a high-level administrator and a frontline worker. Medical students were selected randomly from among the class of first year students. Faculty members were predetermined by their positions of leadership and stewardship over undergraduate medical education, family medicine and social accountability. Agency directors were chosen based on the subgroups of the homeless population served by that agency but not specifically served by the CDIRC (e.g. families) or not a specific focus of the CDIRC (e.g. Aboriginal peoples, and seniors). Clients were recruited via convenience sampling, using the following inclusion criteria: a) homeless for at least one week once in the last 6 months; b) 18 years of age or older; c) speaks and understands English; and d) has no acute mental illness or other condition that may preclude giving informed consent. Over a 5-day period, staff recruited clients who met the criteria. All participants gave informed consent.

A semi-structured format was used for all interviews. Separate interview guides were drafted for each type of stakeholder (as classified above). Participants were asked about issues of relevance to them: providers were asked about their perceptions of healthcare needs of the homeless and what a SRC could to do assist them in meeting these; faculty and students were asked about the role of caring for the underserved and of a SRC may play in the educational curriculum; clients were asked about where they access primary healthcare and barriers they face, as well as about their knowledge of the SRC and its potential strengths and limitations.

Two field researchers (DC & KG) conducted the first three interviews together, with the remainder of individual interviews conducted by either one of the researchers. They worked together for group interviews – one asked questions while the other observed participant interactions and recorded field notes. Neither of these researchers was involved in the provision of clinical services during the time of data collection.

All interviews were recorded digitally and transcribed verbatim. Interview transcripts were analyzed in NVivo 7™ (QSR International), using a coding template devised from the literature. The analysis was iterative and comparative: Each transcript was coded individually and new codes were added to the template, returning to previous interviews to determine if those contained data relevant to the new codes. Codes were organized into categories and then themes [28].

Trustworthiness and credibility [28,30] were achieved by methods of consensus and member-checking. Two researchers coded each transcript and reached consensus on coding. Themes were discussed by the team as interpretation developed. Each participant was asked to provide their feedback on a four-page summary of findings to ensure that perspectives were correctly and appropriately represented.

Ethics approval for the study was granted by the University of Calgary’s Conjoint Health Research Ethics Board.

## Results

Three themes emerged from the data: benefits of the SRC, barriers to the SRC, and future directions.

### Benefits of the SRC

All participants agreed that the SRC had value for serving homeless populations and for medical education purposes. Student providers were thought to have more time to listen and educate patients than physicians – this time may permit development of more empathetic relationships than possible in a typical brief patient-physician encounter.

Some client participants felt students were more sympathetic to their issues, while some physicians were reportedly more judgemental, students were thought to be free of some social biases: “you feel more comfortable ‘cause it’s not that intimidating, you know what I mean, and sometimes [students] are more like open, like the mutual respect”.

Non-client participants thought students, by virtue of their relationships in medical school and access to medical specialists, were in a good position to help make referrals to specialist care and to increase accessibility by accompanying patients to the appointments:

Because they’re currently involved with the medical faculty and connected with the hospital and so on, they can make a call to a specialist and say ‘you know I’ve got this really complex interesting situation, can we get this person in?’ When our clients are hospitalized, those students are able to do some follow up there and sort of connect all the dots.

Having the SRC at the CDIRC increased the existing capacity of the CDIRC medical services. In a three hour period, the SRC comprehensively cared for about six complex patients, two more than the physician would typically see when working without students: “The other piece obviously is that they are able to provide for a sample of our population, a really thorough and meaningful assessment and attention and follow up”.

With the SRC located at the patients’ place of residence (i.e., the CDIRC), it was essentially offering *home* visits, which other healthcare providers for the homeless do not regularly provide. Further, clinic hours were in the evening. This is especially relevant to the ‘working poor’, homeless individuals who work during the day and cannot afford to attend clinics in daytime hours. As one provider said: “Having the service in the CDIRC means that people will access it because it’s not difficult. It’s right there, they don’t have to do anything special, and it’s right in the place they call home”.

The SRC was seen to have an important role within the Faculty of Medicine at the University of Calgary. Faculty stakeholders identified that the SRC contributes to the faculty’s mandate areas of education and service to society, and to its renewed interest in social accountability: “You’re meeting patients, you are giving back to the community, you are using your skills, you’re learning about underprivileged populations, you’re learning about social accountability, and you’re bringing back something to the Faculty”.

Working at the SRC allowed medical students to expand their education, providing opportunity to improve their clinical reasoning and skills of history taking and physical examination. Several providers expressed hope that early exposure to marginalized populations may increase interest and competency in providing care to this population later in their medical career: “The more of those who are in the beginning stages of practice who become aware of and exposed to [this population], the better able they’re going to be to serve [them]”.

### Barriers to the SRC

Perceived barriers related to infrastructure and personnel. While the location of the SRC had its benefits, being in the CDIRC also posed certain challenges. Functioning in this location allowed the SRC to only serve a subset of the homeless population, those familiar or comfortable with the CDIRC. We were told that “families in crisis and families who are homeless have significantly different needs than single men and women”, and that families and seniors would be unlikely to access our clinic at the CDIRC. It was suggested that the SRC be expanded to multiple sites, such as other homeless shelters, in order to meet the needs of various subpopulations who are underrepresented at the CDIRC.

Also related to infrastructure was concern about the ability of the SRC to provide continuity of care to its patients. Several provider participants identified that a clinic model that only functions once per week, and is staffed by different clinicians each week, would not be able to provide the needed continuity: “you’re going to find that because you are rotating through a schedule, you won’t get that same continuity of care. Your communication between yourselves is going to be really important”. The longitudinal relationship family physicians have with their patients cannot be developed by the students working in the SRC. There is an ongoing relationship with the CDIRC attending physician, however, which partially mitigates this concern.

A key barrier, repeatedly described by all participant groups, was the lack of student medical knowledge and experience:

The nature of the medical knowledge that each one of you holds could be an obstacle in that if you don’t interface with each other and supervisors it could be a problem. We all have limited knowledge and we all have to understand what is our scope. That’s a consideration right, not to work beyond your scope and to address it, you need to work in a teams based format amongst the students and work effectively with the staffing that’s there.

Most participants, both providers and clients, were comfortable with the model used by the SRC, which ensured the attending physician saw each patient, reviewed the students’ assessments, and worked with them to develop a treatment plan for the patient.

### Future directions for the SRC

Participants also provided suggestions on how the SRC could be improved moving forward. They recommended communicating effectively with other organizations that serve homeless populations. This was considered especially important to those who provide healthcare as part of their services: “Now is the time to communicate it to your key partners and your key community partners that deal with homeless because the more we know, then the more we can just help you”.

Since the SRC model is not ideally suited to providing longitudinal care, some participants suggested the SRC focus on acute care. Expansion was commonly cited: adding preceptors, sites, or medical specialty clinics.

Service providers and clients perceived that the unmet primary care needs were great amongst Calgary’s homeless populations, as were the barriers that prevent homeless people from accessing the services they require. The SRC was seen as a model with potential to make a significant contribution to the provision of care among this population, while providing an important educational experience for medical students.

## Discussion

This study confirmed that in the context of a large Canadian city, SRCs can play an important role in the provision of primary care for the homeless, offer considerable tangible benefits to patients, and provide a valuable contribution to medical education.

One of the benefits in evaluation planning is identifying inconsistencies, and we would argue, in the case of the SRC, that this is an important aspect of ensuring this program is actually involved in social change that improves the lot of the homeless [8]. What became evident in this study was that several aspects of student-run clinics may help bridge limitations of traditional primary care for this population. When discussion of SRCs draws on models of primary care that focus on aspects such as continuity of care and long-term relationships with family physicians it will miss the obvious fact that people experiencing homelessness have very particular needs that cannot be accommodated by the healthcare system that is in place without some change.

SRCs are not without shortcomings. Using the appropriate model to identify shortcomings can provide adequate foresight and planning to address them and optimize operations, rather than providing decontextualized information that does not facilitate program improvement. In this case, for example, if the SRC was evaluated for outcomes concerning continuity of care it might be found lacking, whereas our results indicate that clients considered it a beneficial resource. We did not assess outcomes in this study, nor did we evaluate the effectiveness of the SRC as an intervention in this population.

Another aspect of primary care raised consistently in this study, which has been previously identified with SRCs in the United States [17] is interprofessional practice. Given the complex health needs of those who access SRCs [14] this appears to be an important aspect of SRCs, with the additional benefit of adding interprofessional educational opportunities [3,17].

Clearly, SRCs can contribute to meeting the complex healthcare needs of underserved populations within urban settings. Lacking from the observations and comments of study participants was the importance of the flexibility, commitment and willingness to train embodied by the attending physician. The training in primary care in a SRC may be at odds with what students receive in medical school concerning matters of continuity, patient compliance, specialization, and so on. SRCs therefore have the potential to change medical education in Canada and we suggest this may become an explicit social change goal.

Our stakeholders’ discussions did not address the need for changes in how the system provided care to homeless people. Rather, participants suggested SRCs be modeled to try to meet all needs. These include extended hours and increased access to primary care physician services, which are deficiencies documented previously in research regarding the Canadian healthcare system [6] but never before assessed in the context of a SRC program. The suggestion that medical students could provide non-judgmental, empathetic care in comparison to care provided by physicians has been demonstrated in the United States [17], but is a novel finding in Canada. It raises the possibility of other students (e.g., nursing, social work) also being trained to do primary care assessments. Two studies [19,20] have shown that students involved in SRCs are more likely to work with underserved populations in their future, but whether long-term interdisciplinary practice [31] is promoted through SRCs is unclear.

This study is, of course, not without limitations. Since the field researchers conducting the interviews were also involved in running the clinic, there is potential for social desirability bias to have influenced respondents. However, they were not performing clinical duties at the time of data collection, and had never had clinical contact with any client participants. Given the scope, we acknowledge that it is a snap-shot in time of access to primary care of a homeless population in one particular socio-political context. We recognize that even in the context of Calgary, additional research may be needed to determine how SRCs can serve families, youth, and Aboriginal people who comprise a large proportion of the homeless population in this setting. Furthermore, this study was not designed to evaluate outcomes or processes of care from the SRC, but simply an exploration of stakeholders’ views on the role of such a clinic.

## Conclusions

SRC programs have been recommended as mandatory components of medical education [32]. It has been well documented that even in a system of publicly funded care, underserved Canadians experience poorer health outcomes than the rest of the population [33]. Our results indicate there are unmet healthcare needs in the context of a large Canadian city that key stakeholders believe a SRC could address. Although there are significant barriers associated with a SRC providing these services, its potential contribution is highly valued by fellow providers, homeless clients and student practitioners alike. Published research is needed that examines Canadian SRC programs to determine what works best and why. This will facilitate optimal planning and maximal benefit to communities where SRCs operate and contribute critical reflection on their roles in the healthcare system.

## Competing interests

Funding for this study was provided by the University of Calgary’s Student Run Clinic. This organization held no influence in any part of the study or manuscript preparation.

Three of the authors were involved in the establishment of the Student Run Clinic program. The fourth author and supervisor of this study are experienced in program evaluation and homelessness research and has no vested interest in the clinic or the CDIRC.

## Authors’ contributions

WET, with expertise in program evaluation and experience in homeless research, contributed substantially to conception and design, and interpretation of data. DJTC and KG contributed to conception and design, data collection, and analysis and interpretation of data. BGO contributed to the interpretation of data, review of the scientific literature, and formulation of the final manuscript. All authors contributed to the article draft and approved the final manuscript.

## Authors’ information

Dave Campbell, MD, MSc is a PhD candidate in Health Services Research (Department of Community Health Sciences), as well as a resident in Internal Medicine (Department of Medicine) at the University of Calgary. Braden O’Neill, BA is a DPhil candidate in Primary Health Care Sciences at the University of Oxford, as well as a MD candidate at the University of Calgary. Katherine Gibson, MD, MPH is a resident in Family Medicine at Queen’s University. Wilfreda Thurston, PhD is a Professor in the Department of Community Health Sciences, Faculty of Medicine and Department of Ecosystem and Public Health, Faculty of Veterinary Medicine at the University of Calgary.

## Pre-publication history

The pre-publication history for this paper can be accessed here:

http://www.biomedcentral.com/1472-6963/13/277/prepub
